# Foreword to the Focus Issue: Composite materials for functional electronic devices

**DOI:** 10.1080/14686996.2022.2126140

**Published:** 2022-09-23

**Authors:** Ye Zhou

**Affiliations:** Institute for Advanced Study, Shenzhen University, Shenzhen 518060, China

Materials are considered the basis for human survival and development, and also the precursor to the development of human society. Nowadays, the technological revolution is developing rapidly, and the pace of industrial upgrading and material replacement is accelerating. The integration of new material technology with nanotechnology, biotechnology, and information technology has become the main direction of materials science and engineering. With the rapid growth of information technology, advanced electronic devices that possess high performances, multi-functions, and high flexibility become highly demanded.

Composite materials are multifunctional material systems, which provide properties that cannot be obtained by any single material. A composite material is a combination of two or more substances with different chemical properties or different organizational phases in a micro or macro form. Composite materials can be mainly divided into two categories: structural composite materials and functional composite materials. Functional composite materials are generally composed of functional body components and matrix components. The matrix not only plays the role of forming the whole but also can synergize or strengthen the function. The functional body may be composed of one or more functional materials. Functional composites refer to composites that provide other physical properties in addition to mechanical properties, for instance, conductive, magnetic and optical properties, etc. Multifunctional composite materials can have multiple functions. At the same time, it is also possible to generate new functions due to compounding effects. Multifunctional composite materials have been the important development direction of functional composite materials.

Due to the wide selectivity of composites-based materials, developing composites-based electronic devices has been one of the best choices to meet the demand for future electronic devices. A series of composite materials with desirable electronic and optical properties has been developed. It is expected that new phenomena of composite materials and devices can be discovered in the future, and more novel mechanisms, structures, and applications will emerge. There still exist challenges in researching functional materials in electronics and optoelectronics.

This Focus Issue on “Composite materials for functional electronic devices” have organized high-quality works and topics focusing on the latest advances in composite materials based on nanomaterials and related nanomaterial systems to understand the electronic and optoelectronic properties and develop functional electronic devices such as sensors, transistors, memories, spintronic devices, solar cells and so on.

The paper by Han et al. from Shenzhen University reviews the synthesis and applications of perovskite quantum dot-based composites in diverse areas such as photonics, electronics, and sensors. They also discuss the strategy of enhancing optoelectronic properties as well as chemical, thermal and photostability of perovskite quantum dot-based composites. The paper by Xu et al. from Soochow University describes the recent progress in polymer-inorganic nanocomposite-based gas sensors. They introduce the roles of inorganic nanomaterials in improving the gas-sensing performances of conducting polymers as well as the development of conducting polymer-inorganic nanocomposites. Furthermore, they present a perspective on gas sensors incorporating polymer-inorganic nanocomposite. The paper by Zhuge et al. from Ningbo Institute of Materials Technology and Engineering, Chinese Academy of Sciences reviews the recent advances in the development of hybrid oxide-based memristive devices. The blending schemes as well as the working mechanisms of hybrid oxide memristors are discussed, and the promising prospects for the application of oxide memristors to neuromorphic computing are presented. The paper by Lin et al. from National Tsing Hua University is a review of the medical applications of piezoelectric and triboelectric nanogenerators in the development of wound healing technology. An outlook on the current challenges and future development in meeting medical needs and commercialization is discussed in detail. The paper by Yu et al. from Shenzhen Institute of Information Technology provides a comprehensive review of the recent advances in the engineering strategies of two-dimensional ferromagnets, such as strain-, doping-, structural- and electric field-engineering. Some useful guidelines for exploring the fundamental physical properties and practical spintronic devices of low-dimensional magnets are also introduced. The paper by Cao et al. from South China University of Technology reviews the recent developments in the interfacial chain assembly of conjugated polymer poly(3-hexylthiophene-2,5-diyl) (P3HT) in the flat/curved confined space and its applications in electronic devices. The interfacial assembly of P3HT at the nanoscale can help to improve the electronic properties of the resulting devices, such as charge transport and power conversion efficiency, giving general implications for designing high-performance electronic devices.

As guest editor, I am in no doubt that this STAM Focus Issue provides very interesting and latest advancements in the science and technology of composite materials for functional electronic devices. I hope this Focus Issue can inspire various striking developments in the field and provide some guidelines for future development of composite materials in electronic devices.

